# Temporal response dynamics of *Drosophila* olfactory sensory neurons depends on receptor type and response polarity

**DOI:** 10.3389/fncel.2012.00054

**Published:** 2012-11-16

**Authors:** Merid N. Getahun, Dieter Wicher, Bill S. Hansson, Shannon B. Olsson

**Affiliations:** Department of Evolutionary Neuroethology, Max Planck Institute for Chemical EcologyJena, Germany

**Keywords:** odorant receptors, ionotropic receptors, pulse resolution, single sensillum recording

## Abstract

Insect olfactory sensory neurons (OSN) express a diverse array of receptors from different protein families, i.e. ionotropic receptors (IR), gustatory receptors (GR) and odorant receptors (OR). It is well known that insects are exposed to a plethora of odor molecules that vary widely in both space and time under turbulent natural conditions. In addition to divergent ligand specificities, these different receptors might also provide an increased range of temporal dynamics and sensitivities for the olfactory system. To test this, we challenged different *Drosophila* OSNs with both varying stimulus durations (10–2000 ms), and repeated stimulus pulses of key ligands at various frequencies (1–10 Hz). Our results show that OR-expressing OSNs responded faster and with higher sensitivity to short stimulations as compared to IR- and Gr21a-expressing OSNs. In addition, OR-expressing OSNs could respond to repeated stimulations of excitatory ligands up to 5 Hz, while IR-expressing OSNs required ~5x longer stimulations and/or higher concentrations to respond to similar stimulus durations and frequencies. Nevertheless, IR-expressing OSNs did not exhibit adaptation to longer stimulations, unlike OR- and Gr21a-OSNs. Both OR- and IR-expressing OSNs were also unable to resolve repeated pulses of inhibitory ligands as fast as excitatory ligands. These differences were independent of the peri-receptor environment in which the receptors were expressed and suggest that the receptor expressed by a given OSN affects both its sensitivity and its response to transient, intermittent chemical stimuli. OR-expressing OSNs are better at resolving low dose, intermittent stimuli, while IR-expressing OSNs respond more accurately to long-lasting odor pulses. This diversity increases the capacity of the insect olfactory system to respond to the diverse spatiotemporal signals in the natural environment.

## Introduction

Insect olfactory sensory neurons (OSN) express a large number of receptor proteins of different types. These receptor types include ionotropic receptors (IR), gustatory receptors (GR), and odorant receptors (OR) (Clyne et al., 1999; Vosshall et al., 1999; Benton et al., 2009). IRs are composed of three trans-membrane proteins and co-receptors, while GRs and ORs are seven trans-membrane proteins (Vosshall et al., 1999; Benton et al., 2006, 2009). ORs are co-expressed with the ubiquitous co-receptor Orco, while Gr21a, a CO_2_ sensor, is co-expressed with Gr63a (Benton et al., 2006; Jones et al., 2007). All OSNs are housed within different morphological types of olfactory hairs, known as sensilla. There appear to be important organizational differences between OSNs that express IRs, GRs, or ORs. Multiple IRs and GRs can be co-expressed per neuron, while OR expression generally follows a one neuron-one receptor rule (Thorne et al., 2004; Wang et al., 2004; Couto et al., 2005; Benton et al., 2009). Receptors from different protein families can also be co-localized in the same sensillum (Couto et al., 2005; Song et al., 2012). For example, in *Drosophila*, the ab1 sensillum houses four OSNs, three expressing ORs and one expressing Gr21a. Also, in the *Drosophila* coeloconic sensillum ac3 an OSN expressing Or35a is co-localized with an OSN expressing Ir75abc (Yao et al., 2005; Silbering et al., 2011).

These diverse receptors have evolved at different points in evolutionary time (Robertson et al., 2003; Croset et al., 2010). Recent research also suggests that many have broad affinity to different chemical classes (Hallem et al., 2004; Yao et al., 2005; Benton et al., 2009; Ai et al., 2010). Yet specificity might not be the only reason for receptor diversification. In the natural environment, insects are constantly challenged with odors not only of diverse molecular types, but with diverse spatio-temporal dynamics. At some distances, odor plumes can present brief and intermittent stimuli (Kaissling et al., 1987; Vickers et al., 2001) with low molecular flux, while at close range or high molecular flux, odors could present a nearly continuous stimulus (Murlis et al., 2000; Louis et al., 2008; Gomez-Marin et al., 2011). These spatiotemporal factors could also be a significant driving force for diversification. The behavior of an insect is a result of the integration of responses from several OSNs expressing a variety of receptor types (Silbering et al., 2011). Thus it is worthwhile to characterize the response dynamics across the OSN repertoire.

To address whether these different receptor types exhibit differences in temporal response kinetics, we assess the response dynamics of *Drosophila* OSNs expressing various receptor types to both different stimulus durations and frequencies. We evaluate the temporal dynamics of antennal OSNs expressing ORs (Or59b and Or35a), IRs (Ir84a, Ir75abc, and Ir41a), and GRs (Gr21a). Or59b-OSNs and Ir41a-OSNs respond with either excitation or inhibition to different ligands, and were chosen to assess the effect of response polarity on temporal kinetics. Or35a- and Ir75abc-OSNs are housed in the same sensillum, and are tested to control for the effects of the perireceptor environment on the temporal response. Finally, Gr21a-expressing OSNs are the only GR-expressing OSNs found on the antenna. Here we show that sensory neurons expressing receptors from different protein families also exhibit different dynamics to brief and intermittent stimuli.

## Materials and methods

Both male and female flies at 2–6 days of age were used. Stocks were maintained on conventional cornmeal agar medium under a 12 h light: 12 h dark cycle at 25°C.

### Electrophysiology

A fly was mounted in a cut pipette tip with the head protruding and small amount of wax placed into the tip end to prevent movement. The pipette was then fixed onto a microscope slide with wax and the antennae fixed on a cover slip with a sharpened glass micropipette, similar to (Hallem et al., 2004; Yao et al., 2005; Pellegrino et al., 2010). An electrolytically sharpened tungsten electrode was placed in the eye for grounding and a sharpened tungsten recording electrode was brought into contact with the base of the sensillum using a Luigs and Neumann, SM-59 manipulator (Ratingen, Germany) at 1000× magnification with an Olympus BX-51 microscope (Olympus Corporation, Tokyo, Japan).

### Odor stimuli

**Figure 1:**
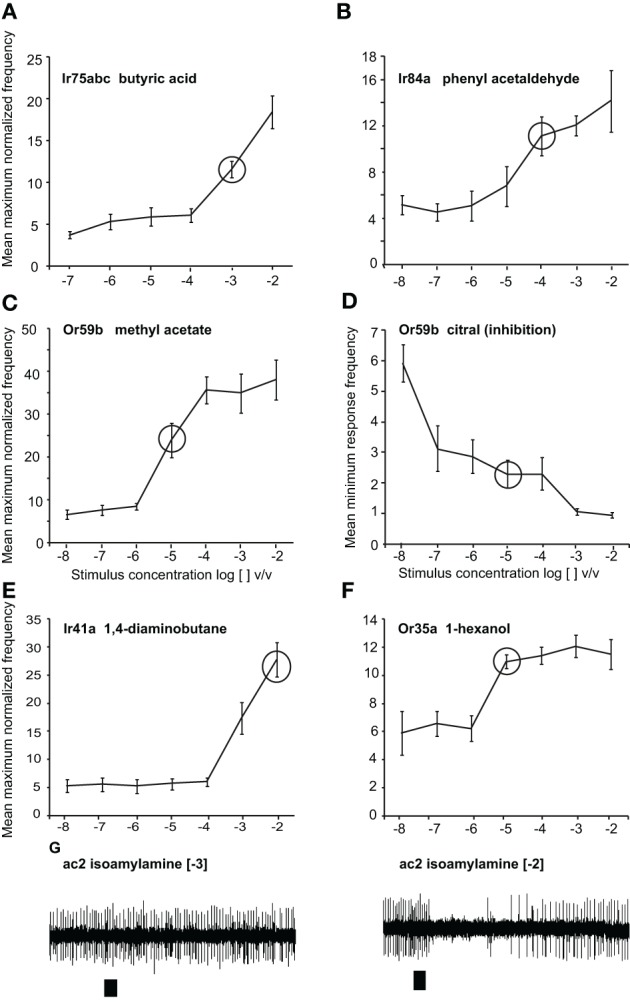
**Responses to odors at different doses.** Dose-response curves presented as normalized maximum frequency response for **(A)** Ir75abc-expressing neurons to butyric acid *n* = 8–13 **(B)** Ir84a-expressing neurons to phenylacetaldehyde, *n* = 9–12. **(C)**, Or59b-expressing neurons to methyl acetate, *n* = 8–17 **(D)** Or59b-expressing neurons to citral presented as the minimum frequency, *n* = 6–10. **(E)** Ir41a-expressing neurons to 1, 4-diaminobutane *n* = 6–8 **(F)**. Or35a-expressing OSNs to 1-hexanol, *n* = 6–8. **(G)** Representative traces showing the response of OSNs of ac2 sensilla to isoamylamine at two different concentrations (responses to lower concentrations were not observed). Please note that while only Ir41a-expressing neurons are excited by 1, 4-diaminobutane in this sensillum (ac2), all neurons are inhibited by isoamylamine, and we thus label the inhibitory responses with the entire sensillum label.

Methyl acetate (>98%), citral (>95%), phenyl acetaldehyde (>90%), butyric acid (>99%), 1, 4-diaminobutane and isoamylamine (>98%), 1-hexanol (>99%), and ethyl hexanoate (>99%) were purchased from Sigma Aldrich Germany. Phenyl acetaldehyde, methyl acetate, 1-hexanol and ethyl hexanoate were diluted in mineral oil (BioChemika Ultra, Fluka), and butyric acid, 1, 4-diaminobutane, and isoamylamine were dissolved in water. Citral was dissolved in hexane (>99%, Fluka Analytical, Buchs, Switzerland). We chose odor concentrations within the linear portion of the dose response curve and the tested concentrations are indicated with circles (Figure 1). All concentrations are reported as log [odor] v/v. For Gr21a stimulation, a 1.5 ml glass vial was filled with pure CO_2_ and placed into the stimulus system similar to the other stimuli. After each frequency set (1–10 Hz), the CO_2_ was refilled.

For frequency stimulation, we used a custom-built multicomponent stimulus system similar to (Olsson et al., 2011). Briefly, 400 ul of appropriate dilutions of each odorant was added to an Eppendorf tube and placed in the bottom of a PEEK vial (4.6 cm × 2.5 cm × 2.5 cm dimensions). Each vial was sealed with a stainless steel plug (Olsson et al., 2011). The pulse duration, inter-stimulus interval and number of pulses were adjusted through a custom built Labview program (Olsson et al., 2011). The odors were delivered from the headspace via Teflon tubing 150 cm long with an inner diameter of 1 mm and positioned as close as possible (~1.5 cm) to the antennae. The flow rate of air was 0.5 L/min. For stimulation, the stimulus system was connected to the IDAC (Syntech, Ockenfels, Germany) and through USB connection to a PC. Stimulation was controlled by an OEM (EDP 0504, thinXXS) pump control system and DAQ (USB 6008 data acquisition hardware, National Instruments, Austin, TX, USA) with custom-built Labview 8.5 software (built by Daniel Veit; National Instruments). For frequency stimulation the on time was 50 ms for OR- and Gr21a-OSNs and the off time was adjusted from 950 ms or 50 ms for 1–10 Hz, respectively. For IR-OSNs stimulated with [−4] and [−3] stimulus concentrations, the pump on time was 200 ms and off time 800, 300, or 50 ms for 1–4 Hz, respectively. At [−2], the protocol was identical to the OR-OSNs and Gr21a-OSNs. The consistency of odor delivery for different pulse durations and frequencies was confirmed using PID (200a, Aurora Scientific Ontario, Canada).

### Data analysis

All raw spike data were acquired and converted to digital spikes using Autospike 3.7 (Syntech). Co-localized neurons were identified based on spike amplitude. Peri-stimulus time histograms (PSTHs) were obtained by averaging spike activities in 25 ms bins from the start of the stimulation and normalized to the average frequency for 2 s before stimulation (Olsson et al., 2011; Sargsyan et al., 2011). The OSN responses between consecutive pulses were compared using repeated measure ANOVA by assessing the normalized mean of area under curve (AUC) spike frequency per each stimulus duration, i.e. pump on time + off time. Consecutive pulses were normalized to the response of 1st pulse. Between treatments, a Mann-Whitney U test or *t-test* was used depending on the normality of the data. To evaluate the capacity of receptors to resolve pulsed stimuli, we visualized the response using normalized peri-stimulus histograms and quantified the % return to the spontaneous activity (baseline), using the ratio between the first value in the 2nd pulse and the maximum peak value of the first (previous) pulse converted to a percentage: Percent return to baseline = 1−(1st value of the 2nd pulse/maximum frequency of the 1st pulse) × 100 (Bau et al., 2002). A One-Way ANOVA followed by a Tukey *post-hoc* test was performed to determine if the return to baseline was significantly reduced between the different stimulation frequencies. Latency was measured as the time from the onset of the odor stimulus to the maximum response frequency (mechanical delay was not considered). Response width was calculated as the time between half-maximal response for excitation and half-minimal response in the case of inhibition. Spearman's correlation was used to assess the relationship between repeated pulses and latency as well as between response width and intensity with stimulus duration. All analyses were performed using SPSS version 17 (IBM Corporation, Armonk, New York, US).

## Results

### Response dynamics of different sensory neurons to varying stimulus durations

**Figure 2:**
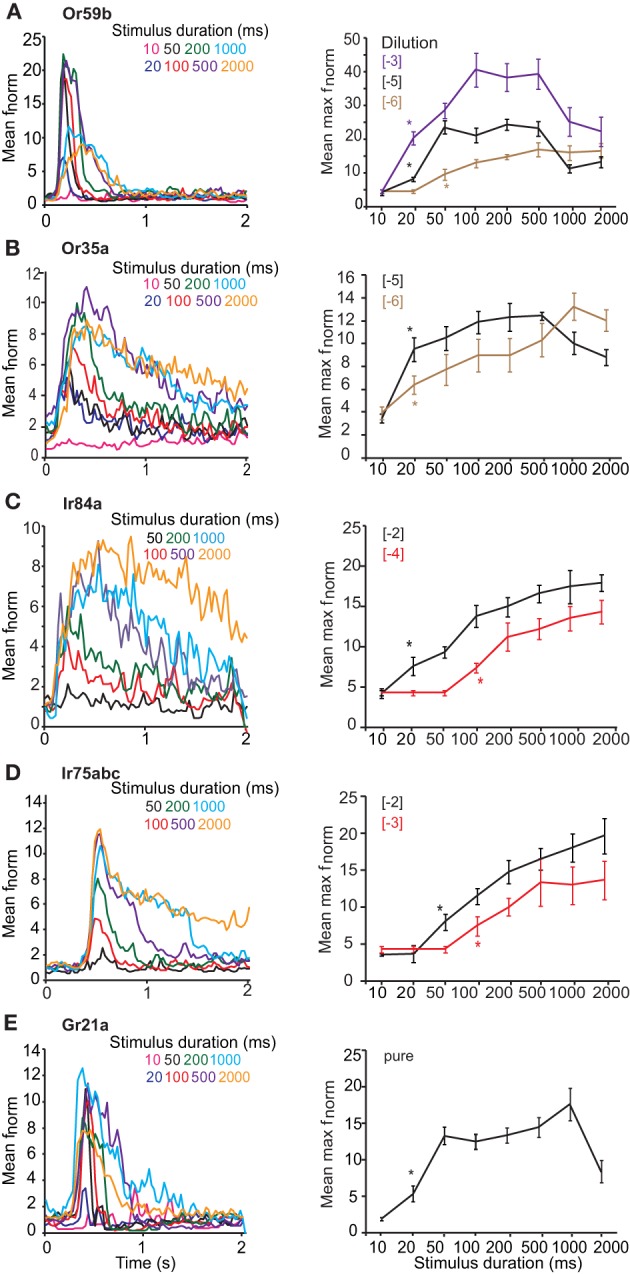
**Response of OSNs to varying stimulus durations.** (**A**, left) Mean peri-stimulus time histograms (PSTHs, 25 ms bins) showing the response of Or59b-expressing OSNs to various stimulus durations of log [−5] v/v methyl acetate. (**A**, right) Mean normalized maximum frequency for Or59b-expressing neurons plotted vs. stimulus duration (*n* = 8–15) for three different concentrations. Asterisks indicate the minimum stimulus duration that elicited a significant response, *P* < 0.05. (**B**, left) Mean peri-stimulus time histograms as in **(A)** showing the response of Or35a-expressing OSNs to various stimulus durations of log [−5] v/v 1-hexanol. (**B**, right) Mean normalized maximum frequency for Or35a-expressing neurons plotted versus stimulus duration for log [−5] and [−6] v/v of 1-hexanol (*n* = 6–14). (**C**, left) Response of Ir84a-expressing neurons to various durations of log [−4] v/v phenyl acetaldehyde as in **(A)**, *n* = 8–10 (**C**, right) as in A for two different concentrations. (**D**, left) Response of Ir75abc-expressing neurons to various durations of log [−3] v/v butyric acid (*n* = 6–15) and **(D**, right) as in **(C)**. **(E)** Response of Gr21a-expressing neurons to pure CO_2_ at different stimulus durations (*n* = 6–10).

We first assessed the response of OSNs carrying ORs, Gr21a, or IRs to key ligands presented with varying stimulus durations at concentrations found in the linear portion of the dose-response curve for each OSN (Figure 1). OSNs expressing Or59b housed in basiconic sensillum type ab2 were stimulated with methyl acetate at [−5] concentration, with stimulus durations varying from 10 ms to 2 s. At 20 ms, the mean normalized frequency of Or59b-expressing OSNs was greater than the spontaneous activity (*t* = 3.482, *P* = 0.005), indicating that a 20 ms stimulation was sufficient to elicit a response (Figure 2A asterisk right). A maximal stimulus response was obtained with a 50 ms stimulation (*P* < 0.05), however, stimulations of 1 s or more significantly reduced the OSN response maximum (*t* = 3.482, *P* = 0.005, mean normalized maximum frequency for 500 ms vs. 1 s stimulation and *t* = 5.047, *P* < 0.001 for 500 ms vs. 2 s, Student's *t*-test). Similar response dynamics were observed in Or35a-expressing OSNs (*t* = 5.007, *P* < 0.001 mean normalized maximum frequency for 500 ms vs. 2 s stimulation; Figure 2B). Adaptation to long stimulus durations (>1 s) was also apparent for Or22a-OSNs (data not shown). There was also a positive and significant correlation between response width at half-maximal response and stimulus duration for both OR-expressing OSNs (*r* = 0.853, *P* < 0.001 for Or59b-OSNs and *r* = 0.93, *P* < 0.001 for Or35a-OSNs; both Spearman's correlation Figures 2A,B left panels).

OSN expressing Ir84a (Figure 2C) were stimulated with [−4] phenyl acetaldehyde and a significant response was obtained at 100 ms (*P* < 0.001, Mann–Whitney *U*-test; Figure 2B). A maximal response was reached at 500 ms (*P* = 0.001, Mann–Whitney *U*-test, as compared to 100 ms), and the maximum response intensity did not decrease at longer stimulation durations (*t* = 0.605, *P* = 0.554 at 500 ms stimulation vs. 1 s, and *t* = 0.394, *P* = 0.699 for 500 ms vs. 2 s; Student's *t*-test). The response of Ir75abc-expressing neurons was similar when stimulated with [−3] butyric acid, (significant response at 100 ms; Mann–Whitney U, *P* = 0.016, Figure 2D), and reached a maximum response at 500 ms (*t* = 2.286, *P* = 0.036 compared to 100 ms). Furthermore, the response did not change at longer stimulus durations (*t* = 0.096, *P* = 0.924, 500 ms vs. 1 s, *t* = 0.068, *P* = 0.946, 500 ms vs. 2 s; Figure 2D right panel). There was also a positive and significant correlation between stimulus duration and response width at half maximal response (*r* = 0.905, *P* < 0.001 for Ir84a-OSNs, and *r* = 0.917, *P* < 0.001 for Ir75abc-OSNs, Spearman's correlation; Figures 2C and D left panel). Similarly, the Ir41a-OSN response to 1,4-diaminobutane at [−2] did not show adaptation at longer stimulus durations (*t* = 0.073, *P* = 0.944 for 500 ms vs. 1 s stimulations; *t* = 0.01, *P* = 0.992 for 500 ms vs. 2 s stimulations).

OSNs expressing Gr21a, which are housed in ab1 sensilla on the *Drosophila* antenna, respond to pure CO_2_ beginning at a 20 ms stimulation (Mann–Whitney *U*-test, *P* = 0.009 Figure 2E). Peak response was obtained at 1 s (*t* = 4.641, *P* = 0.002, Student's *t*-test compared to 20 ms), while at a 2 s stimulation the maximum response frequency decreased significantly (*t* = 2.63, *P* = 0.02, Student's *t*-test, 1 s vs. 2 s). However, the response latency also became shorter with stimulus duration, decreasing from the 20 ms duration (with a mean half-maximal response on set time of 400 ± 26.35 ms), to 1 s (with a mean half maximal response on set time 300 ± 17.67 ms, *t* = 3.028, *P* = 0.016, Student's *t*-test; Figure 2E left panel). This is opposite to both OR- and IR-expressing OSNs, where there was no difference (Figures 2A–D). Similarly, the response width also increased with stimulus duration (*r* = 0.781, *P* < 0.001, Spearman's correlation, Figure 2E left panel).

Increasing stimulus concentrations reduced the duration required to elicit a response regardless of the receptor expressed. For example, Or59b-OSNs required 50 ms at [−6] to elicit a significant response (*t* = 2.486, *P* = 0.025; Figure 1A right), but only 20 ms at [−3] (*P* < 0.001, Mann–Whitney *U*-test, asterisk in Figure 2A right). Similarly, Ir84a-expressing OSNs stimulated with phenyl acetaldehyde at [−2] required only 20 ms to elicit a significant response (Mann–Whitney U, *P* = 0.02, Figure 2C), while Ir75abc-expressing OSNs required a 50 ms stimulation when the concentration of butyric acid increased by 10× [−2] (Mann–Whitney U, *P* = 0.002, Figure 2D, asterisk right).

However, the dose-dependency of OSN adaptation to long stimulus durations was dependent on the receptor expressed. At [−6] long stimulus durations did not reduce the response of Or59b-expressing OSNs (*t* = 0.292, *P* = 0.776 for 500 ms vs. 1 s; *t* = 0.33, *P* = 0.745 for 500 ms vs. 2 s) or Or35a-expressing OSNs (*t* = 1.151, *P* = 0.147 for 500 ms vs. 1 s; *t* = 0.948, *P* = 0.356 for 500 ms vs. 2 s; Figures 2A,B right). However, at [−3] concentration, stimulations of 1 s or more significantly reduced the Or59b-expressing OSN response maximum (*t* = 2.235, *P* = 0.045 for 500 ms vs. 1 s; *t* = 2.658, *P* = 0.021 for 500 ms vs. 2 s, Figure 2A). In contrast, longer stimulus durations did not reduce the response of IR-expressing OSNs regardless of concentration (Ir84a-expressing OSNs at [−2]: Mann–Whitney U, *P* = 0.847 for 500 ms vs. 1 s; Ir75abc-expressing OSNs at [−2]: *t* = 0.644, *P* = 0.531 for 500 ms vs. 1 s; Ir41a-expressing OSNs at [−2], *t* = 0.073, *P* = 0.944 for 500 ms vs. 1 s; Figures 2C,D right panels).

### Pulse resolution of different sensory neurons

**Figure 3:**
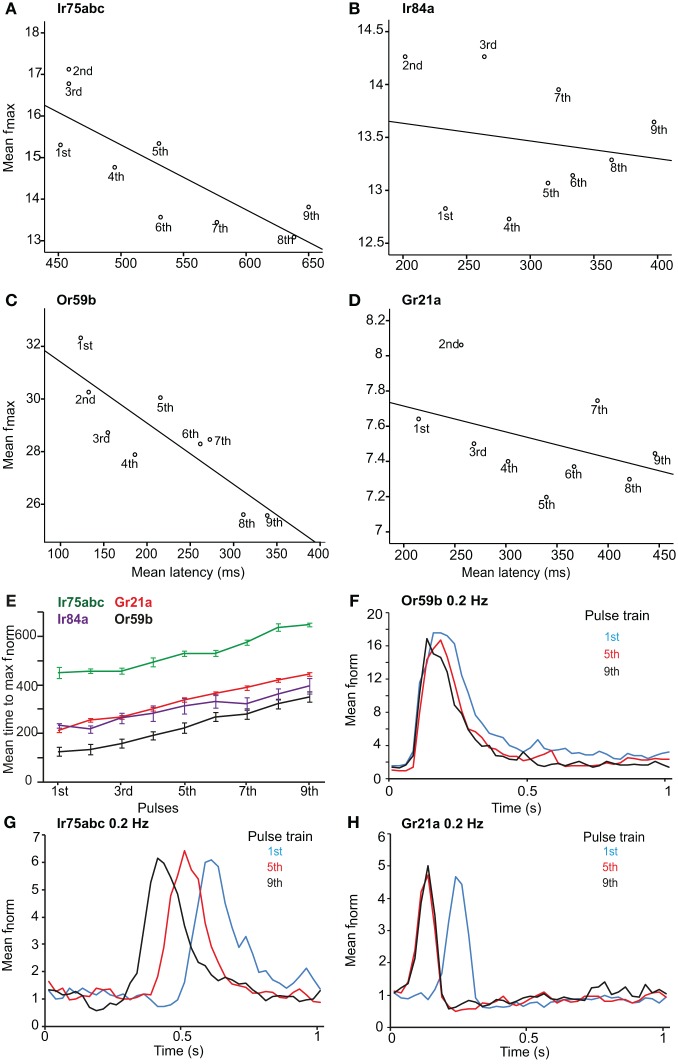
**Latency and maximum response of OSNs to repeated stimuli.** Maximum response frequency vs. time to peak (latency), with best fit line, for OSNs carrying various receptors in response to repeated 1 Hz stimulations. **(A)** Ir75abc, **(B)** Ir84a, **(C)** Or59b and **(D)** Gr21a. Pulse number (1–9) indicated below each point. **(E)** Mean time to maximum response frequency for neurons in **(A–D)** at a 1 Hz repeated stimulation. **(F)** The response onset recovery of Or59b-expressing OSNs when stimulated at 0.2 Hz, *n* = 10 **(G)** as in **(F)** for Ir75abc-expressing OSNs, *n* = 7 and **(H)** as in **(F)** for Gr21a-expressing OSNs, *n* = 10.

After investigating the response of OSNs to various stimulus durations, we presented the neurons with repeated stimulations of varying frequency. The latency to repeated stimulations at 1 Hz increased for all OSN types (*r* = 0.742, *P* < 0.001 for Or59b-OSNs; *r* = 0.94, *P* < 0.001 for Gr21a-OSNs; *r* = 0.787, *P* < 0.001 for Ir75abc-OSNs; *r* = 0.652, *P* < 0.001 for Ir84a-OSNs; Spearman's correlation; Figures 3A–E). However, a variability in latency was observed between the tested OSNs; e.g., Ir75abc-OSNs showed more delayed time to maximum than all other neurons tested, *P* < 0.001, ANOVA followed by Tukey *post-hoc* test (Figure 3E). At 100× stimulus concentrations or a 5 s interstimilus interval, the latency for Or59b-expressing OSNs did not change with repeated stimulation (*r* = 0.09, *P* = 0.475; *r* = −0.006, *P* = 0.952, respectively, Spearmans's correlation; Figure 3F). Similarly, Ir75abc-expressing OSN response onset recovered with a higher concentration (*r* = 0.01, *P* = 0.90, Spearmans's correlation). However, at 5 s interstimulus intervals the response onset became significantly faster for the later pulses (*r* = −0.885, *P* < 0.001, Spearmans's correlation; Figure 3G). The latency also decreased with subsequent stimulations of CO_2_ for Gr21a-expressing OSNs at 5 s interstimulus intervals (*r* = −0.976, *P* < 0.001, Spearmans's correlation; Figure 3H). In summary, this shows that changes in response onset kinetics to repeated stimuli are similar across all tested OSNs and response latencies can be regulated either by altering stimulus concentrations or inter-stimulus intrervals.

**Figure 4:**
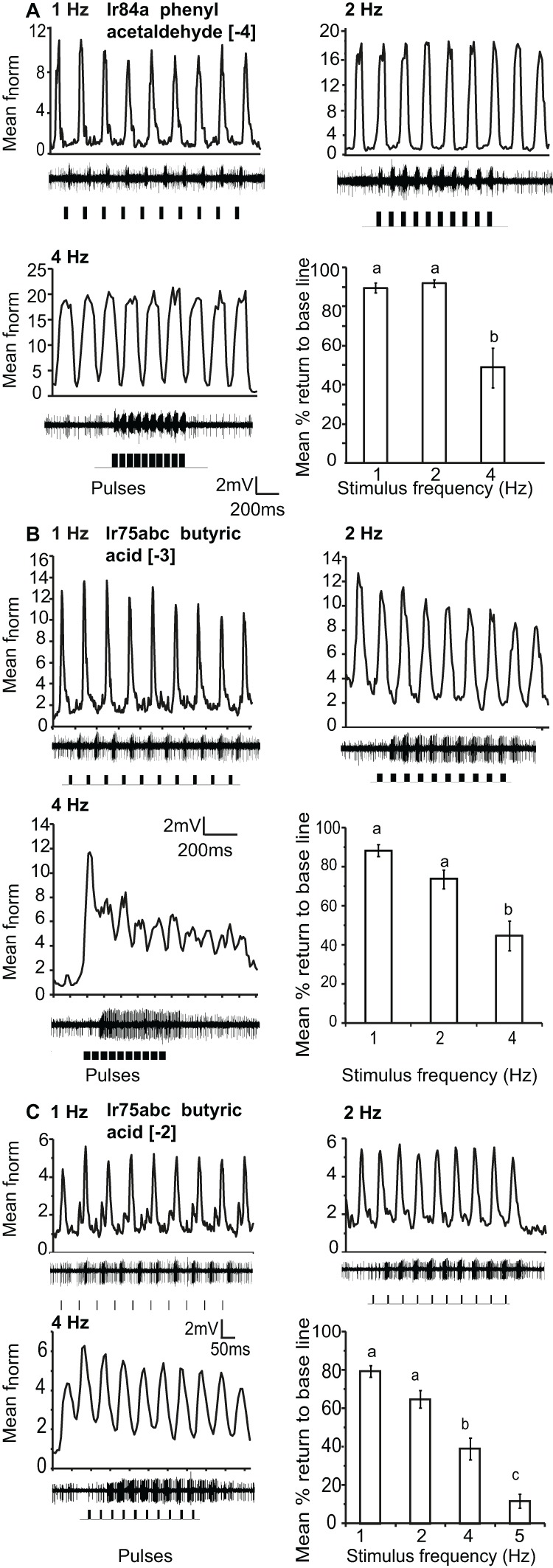
**Response of OSNs to repeated stimulus pulses at varying frequencies. (A)** Average normalized PSTH responses for Ir84a-expressing neurons in response to repeated pulses of log [−4] v/v phenyl acetaldehyde at listed frequencies. Traces below each panel show sample 200 ms recordings. Square pulses indicate stimulus presentation. The final panel shows the mean percent return to base line across all pulses at listed frequencies; error bars indicate SEM (ANOVA, *P* < 0.05, followed by Tukey *post-hoc*, *n* = 7–9). **(B)** Response of Ir75abc-expressing neurons to repeated stimulations of log [−3] v/v butyric acid stimulation as in **(A)** (ANOVA, *P* < 0.05, followed by Tukey *post-hoc* (*n* = 8–10). **(C)** Response as in **(B)** to a 10× concentration of butyric acid (log [−2]); ANOVA, *P* < 0.05, followed by Tukey *post-hoc*, *n* = 14–15).

Ir84a and Ir75abc-OSNs, housed in ac4 and ac3 sensilla respectively, could resolve repeated 200 ms pulses of [−4] and [−3] stimulus concentrations, respectively, up to 4 Hz (the maximum testable frequency due to stimulation length). The mean return to base line during repeated stimulation was significantly reduced at 4 Hz as compared to 1 and 2 Hz stimulation, (*P* < 0.05 ANOVA followed by Tukey *post-hoc* test; Figures 4A,B). At an increased concentration of [−2], Ir75abc OSNs could resolve pulsed stimuli up to 5 Hz at a 50 ms pulse duration (Figure 4C).

**Figure 5:**
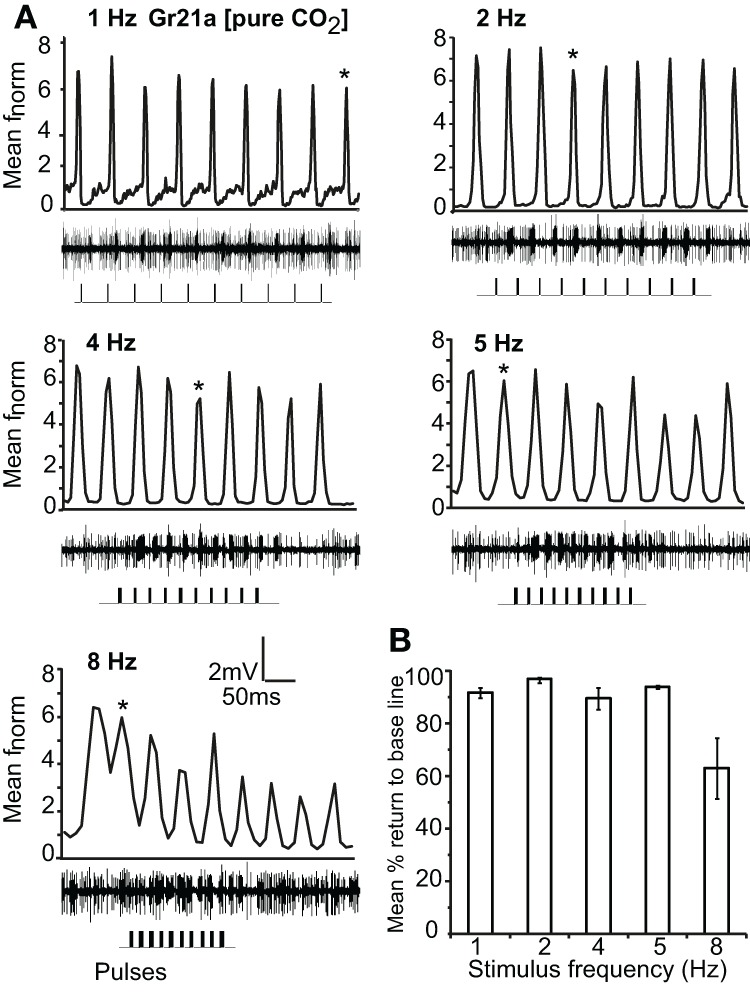
**Response of Gr21a-expressing OSNs to repeated stimulus pulses at varying frequencies. (A)** Average normalized PSTH responses of Gr21a-expressing neurons to repeated pulses of pure CO_2_ at listed frequencies. Traces below each panel show sample 50 ms recordings. Square pulses indicate stimulus presentation. **(B)** Mean percent return to base line across all pulses of listed frequencies, error bars indicate SEM (ANOVA, *P* < 0.05, followed by Tukey *post-hoc* test, *n* = 11–12).

Gr21a-expressing OSNs housed in ab1 sensilla resolved intermittent pulses of CO_2_ as fast as 8 Hz with no significant difference in return to baseline between 1 Hz and 5 Hz stimulations. At 8 Hz, the mean return to base line was significantly reduced, and at 10 Hz only 2.4% recovery to the base line occurred (*P* < 0.001, ANOVA followed by Tukey *post-hoc* test; Figures 5A and B). Gr21a-expressing OSNs also exhibited short term adaptation based on AUC (see “Materials and methods”) that was frequency dependent, i.e. at 1 Hz stimulation the 9th pulse resulted in a significantly reduced response compared to the 1st pulse (repeated measure ANOVA, *P* < 0.001), while at 2 Hz the 4th pulse was reduced (*P* = 0.039), at 4 Hz the 5th (*P* = 0.001), and at 5 and 8 Hz the 2nd (*P* < 0.01, repeated measure ANOVA; Figure 5 asterisks).

### Pulse resolution of stimuli eliciting opposite response polarity

**Figure 6:**
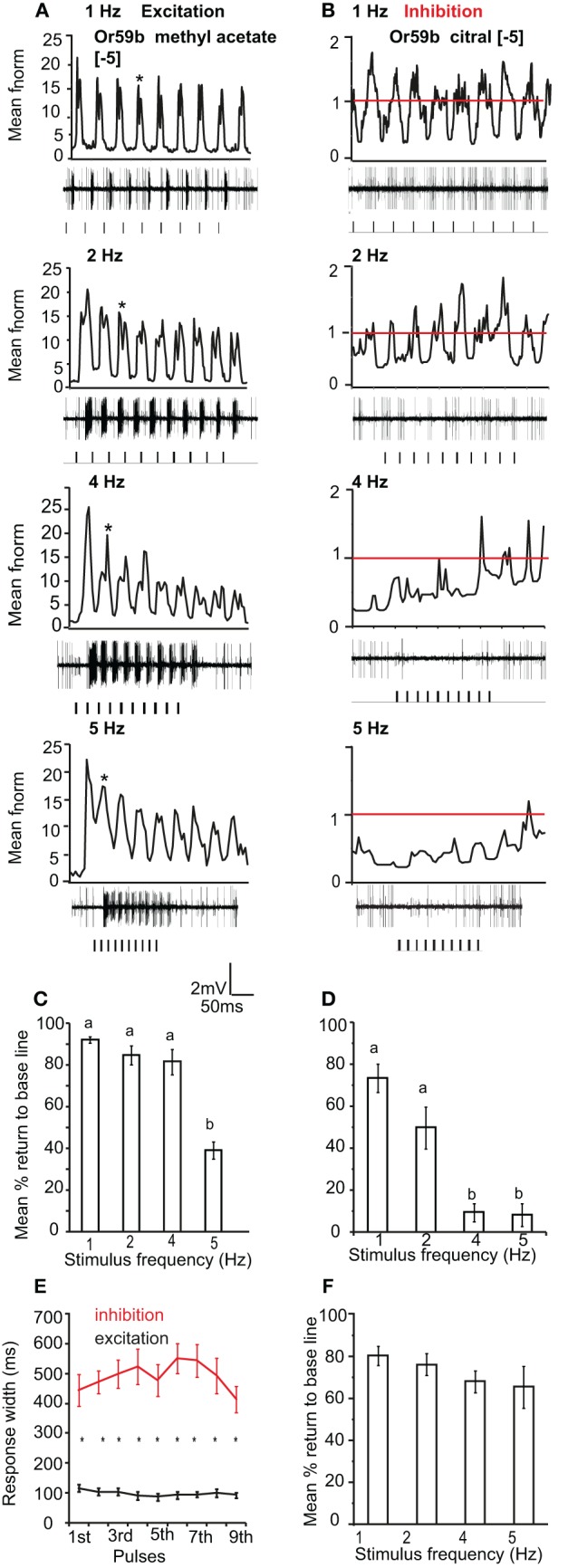
**OR-expressing OSN response polarity and pulse resolution. (A)** Mean normalized PSTH response of Or59b-expressing OSNs to repeated pulses of log [−5] v/v methyl acetate at listed frequencies. Traces below each panel show sample 50 ms recordings. Square pulses indicate stimulus presentation. **(B)** Mean normalized PSTH response of Or59b-expressing OSNs to repeated pulses of log [−5] v/v citral (an inhibitory odor) at listed frequencies as in **(A)**. Red line indicates baseline frequency. **(C)** Mean percent return to base line across all pulses for Or59b-OSN response to methyl acetate, error bars indicate SEM (ANOVA, *P* < 0.05, followed by Tukey *post-hoc* test, *n* = 9–13) and **(D)** as in **(C)** for citral (ANOVA, *P* < 0.05, followed by Tukey *post-hoc* test, *n* = 13–15). **(E)** Mean response width of Or59b-expressing OSNs for excitation and inhibition. **(F)** Mean percent return to base line in response to a pulsed binary mixture of methyl acetate and citral, error bars indicate SEM, (*P* > 0.05 ANOVA, *n* = 8–9).

We also tested the pulse following capacity to single excitatory and inhibitory odor ligands in Or59b-expressing OSNs. We applied [−5] methyl acetate as an excitatory and [−5] citral as an inhibitory ligand. Or59b-expressing OSNs could resolve the excitatory stimulus up to 5 Hz (Figure 6A). The mean return to base line was significantly reduced at 5 Hz stimulation as compared to 1 and 2 Hz (*P* < 0.05, ANOVA followed by Tukey *post-hoc* test; Figure 6C). However, the pulse resolution was also affected by concentration, as a 100× increase in concentration reduced the pulse resolution to 2 Hz (*P* < 0.05). In contrast to the excitatory responses, Or59b-cells were able to resolve pulses of the inhibitory ligand citral only up to 2 Hz, and at 4 and 5 Hz the OSNs showed total inhibition and did not recover when stimulated repeatedly with the inhibitory ligand (*P* < 0.05 ANOVA followed by Tukey *post-hoc* test; Figures 6B and D). The inhibitory ligand also resulted in a larger response width as compared to the excitatory ligand, even though both ligand concentrations were at similar points in the dose response curve (see Figure 1). This indicates that a given OSN response to an inhibitory or excitatory ligand can differ not only in polarity but also in temporal dynamics (Figure 6E). Furthermore, Or59b-OSNs showed short-term adaptation to the excitatory ligand that was frequency dependent (repeated measure ANOVA, *P* < 0.05). At increasing frequencies, short-term adaptation occurred earlier in the stimulus train (Figure 6A asterisk). In contrast, we did not find short-term adaptation based on response width to the inhibitory ligand (repeated measure ANOVA, *P* > 0.05).

**Figure 7:**
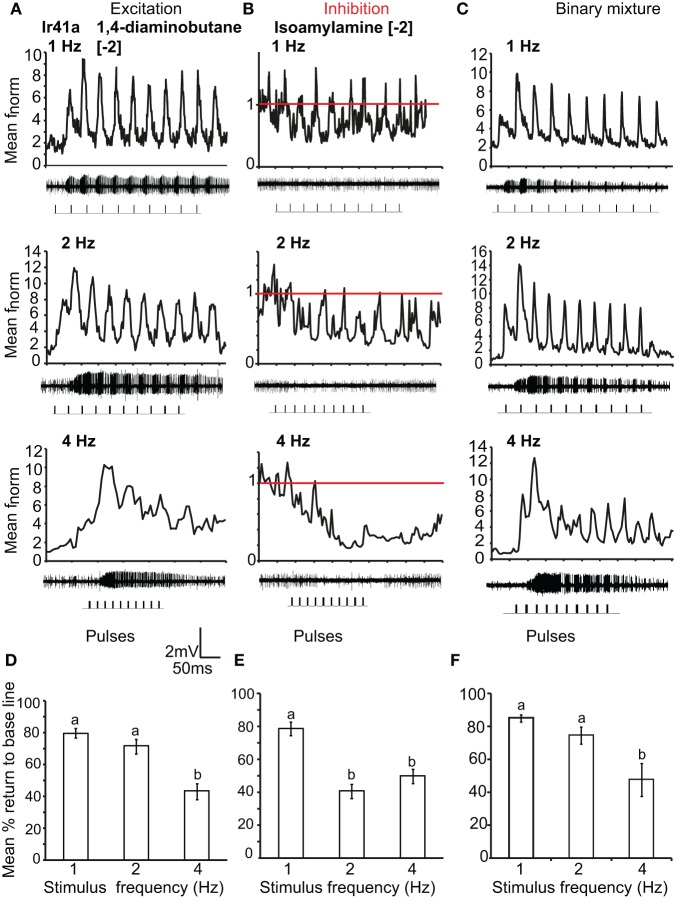
**IR-expressing OSN response polarity and pulse resolution. (A)** Mean normalized PSTH response of Ir41a-expressing OSNs to repeated pulses of log [−2] v/v 1, 4-diaminobutane at listed frequencies. Traces below each panel show sample 50 ms recordings. Square pulses indicate stimulus presentation. **(B)** As in **(A)** for log [−2] v/v of the inhibitory odor isoamyl amine. Red line indicates baseline frequency. **(C)** Mean normalized PSTH response of Ir41a-expressing OSNs to a binary mixture of 1, 4-diaminobutane and isoamyl amine at [−2] v/v. **(D)** Mean percent return to base line to the excitatory ligand across all pulses at listed frequencies, error bars indicate SEM (ANOVA, *P* < 0.05, followed by Tukey *post-hoc* test, *n* = 7–8) **(E)**, as in **(D)** for log [−2] v/v of the inhibitory odor isoamyl amine (ANOVA, *P* < 0.05, followed by Tukey *post-hoc* test; *n* = 7–9). **(F)** as in **(D)** for the binary mixture (ANOVA, *P* < 0.05, followed by Tukey *post-hoc* test, *n* = 7–9).

We also asked if the total inhibition of the neuron at high frequencies of citral (>4 Hz) could interfere with odor coding of the excitatory ligand when presented simultaneously to the OSN. We thus stimulated the neurons with the binary mixture of the two ligands at the concentrations listed above. Stimulation with the two component blend resulted in an improved pulse resolution over either separate odor, with no significant difference in pulse resolution between 1 and 5 Hz (*P* > 0.05 ANOVA (Figure 6F). The effect of response polarity on pulse resolution was also observed in OSNs that express IRs. Ir41a-OSNs exhibited an excitatory response to 50 ms pulses of [−2] 1,4-diaminobutane and resolved pulsed stimuli as fast as 2 Hz, (ANOVA, *P* < 0.05; Figures 7A and D). However, the pulse resolution to the inhibitory ligand isoamylamine at [−2] (the concentration at which the neurons are inhibited by the ligand, Figure 1G) was only maintained at 1 Hz (ANOVA *P* < 0.05; Figures 7B and E). In addition, the binary mixture of 1, 4-diaminobutane and isoamylamine at the same concentration [−2], sharpened the response of Ir41a-OSNs especially at 4 Hz (Figures 7C and F).

## Discussion

Odor stimuli contain three elements of information: odor identity; odor intensity, and a temporal component (Hallem et al., 2004). To respond to these stimuli, insect OSNs express a wide variety of receptors. Here we investigate the response dynamics of OSNs expressing receptors from different protein families to stimuli of both different durations and frequencies. We find that ORs, IRs, and Gr21a exhibit distinct response characteristics that could increase the response range of the insect to the temporally dynamic natural odor environment.

### Response dynamics to different stimulus durations are a function of receptor type

We found that the response of *Drosophila* OSNs to varying stimulus durations (Figure 2) depends on the type of receptor expressed in that neuron. OR-expressing OSNs showed adaptation to higher concentrations of long stimulus pulses (>1 s), both in maximum frequency and latency. This response feature was also independent of ligand (data not shown). In contrast, when IR-expressing OSNs were tested with the same protocol, they required longer stimulus durations to respond, and there was no desensitization even up to 2 s stimulation either in response intensity or latency regardless of stimulus concentration. As a consequence, OSNs that express IRs are able to transmit information concerning the presence of long-lasting odors in their environment better than OR-expressing OSNs. However, this could also present a trade off, because the signal transduction in these OSNs appears to be slower, as seen in Figure 3E, where the time to maximum frequency was longer in IR-expressing OSNs as compared to OR-expressing OSNs.

The difference in response between IR- and OR-expressing OSNs to longer pulses was not a function of stimulus presentation, which was assessed by PID (see “Materials and methods”). It is therefore a property of the OSNs themselves. Are these differences a function of the peri-receptor environment, or rather a property of internal OSN kinetics? To test this, we assessed the response of Or35a-OSNs, which are housed in coeloconic sensillum ac3 together with Ir75abc-OSNs. As with other OR-expressing OSNs, Or35a-OSNs also responded to stimulations as brief as 20 ms and showed desensitization at longer pulses (2 s) in maximum response frequency (Figure 2B). The response kinetics of these OSNs is therefore less influenced by the environment where they are expressed and rather by intrinsic properties of the neurons themselves.

The broad protostome conservation of IRs contrasts sharply with the restriction of ORs to insect genomes. This phylogenetic evidence suggests that IRs were the first olfactory receptor repertoire in insects (Robertson et al., 2003; Croset et al., 2010). IRs are also restricted to coeloconic sensilla, whereas ORs are found in several morphological sensillum types (Gupta and Rodrigues, 1997; Goulding et al., 2000; zur Lage et al., 2003; Benton et al., 2009). Our results show that IR-expressing OSNs required longer stimulation times to respond to key odorants, and responded with lower response intensities. This could imply that IRs are less efficient and less sensitive in detecting and transducing a chemical signal. OR activation results in both ionotropic and metabotropic signaling (Wicher et al., 2008; Deng et al., 2011), while IRs are thought to be purely ionotropic (Benton et al., 2009). Iontropic signaling is also known to be less sensitive (Sato et al., 2008, 2011; Wicher et al., 2008). The requirement for higher concentrations in IR-expressing OSNs has been also shown in Yao et al. (2005). The signal transduction in Gr21a has been shown to involve Gα_q_ protein, but not Gα_s_ (Yao and Carlson, 2010; Deng et al., 2011). Thus, it is possible that the transduction cascade itself leads to these differences in response to varying stimulus durations.

The desensitization/adaptation at longer stimulus durations could affect the temporal accuracy of OR-expressing OSNs in reporting long-lasting odor strands, but it may also enrich the coding possibilities for odor discrimination (DeBruyne and Baker, 2008; Nagel and Wilson, 2011) by allowing the neuron to return to its resting state more quickly. This could provide additional possibilities for odor discrimination such as under background odor, or for resolution of intermittent pulsed stimuli. Adaptation extends the operating range of sensory systems, in some cases over an enormous span of stimulus intensities (Torre et al., 1995). It may also play a role in complex functions of neuronal systems such as stimulus location (Kaissling et al., 1987). Similar results were reported in the locust where the electrophysiological response of projection neurons also depended on stimulus duration (Brown et al., 2005; Mazor and Laurent, 2005). In contrast, the long-lasting response of IR-expressing OSNs could allow for close range detection while on or very near the stimulus source where stimulus durations could persist for much longer periods of time (Murlis et al., 2000; Louis et al., 2008; Gomez-Marin et al., 2011).

### Pulse resolution is receptor type dependent

The different classes of OSNs also showed differences in their pulse resolution to repeated stimuli. Brief intermittent stimuli were not detected by IR-expressing OSNs, in contrast to those expressing ORs (which could respond up to 5 Hz). This response characteristic was mainly due to a difference in sensitivity, as increasing the stimulus concentration for IR-expressing OSNs improved the detection and resolution to 5 Hz. In contrast, a 100× increase in concentration actually reduced the OR-OSNs pulse resolution. The accuracy of encoding rapidly fluctuating intermittent odorant stimuli above 5 Hz was significantly reduced for all OSNs regardless of receptor type. Similarly, other insects resolved up to 5 Hz pulses of general odors or pheromones (e.g., Lemon and Getz, 1997; Barrozo and Kaissling, 2002; Bau et al., 2002), even at the antennal lobe (e.g., Christensen and Hildebrand, 1997; Lei and Hansson, 1999; Lei et al., 2009).

Short term adaptation and latency to peak response to repeated stimuli were independent of the receptor expressed in the OSN (Figures 3A–E). In addition, the time to peak response and the response intensity were recovered in all OSNs either by increasing the inter-stimulus interval to 5 s or by increasing the concentration. This suggests that adaptation to repeated stimulation is a general feature of all OSNs, regardless of the receptor expressed. Adaptation is assumed to be an early step in information processing and decision making (Kaissling et al., 1987; Baker et al., 1988; Dolzer et al., 2003; Theodoni et al., 2011), and appears to affect the response of all OSN types in a similar manner.

### Response polarity affects pulse resolution

Both OR- and IR-expressing OSNs were unable to resolve pulsed inhibitory ligands at frequencies as high as excitatory ligands (Figures 6B and 7B). This could be because the response inhibition lasted longer than excitation (Figure 6E), even though the concentrations tested were at the same point in the dose response curve (Figure 1). According to Ghatpande and Reisert (2011), fast response termination improves pulse resolution. Similarly, Su et al. (2011) showed that the inhibitory responses of OSNs lasted much longer than their excitatory responses, but the reason for this difference is not clear. Interestingly, a mixture of both excitatory and inhibitory odors improved pulse resolution at high frequencies (Figures 6F and 7F). As a consequence, OSNs may respond to intermittent blends at faster rates, which may increase their ability to track complex natural stimuli.

The fast-terminating biphasic response exhibited by Gr21a-OSNs in response to CO_2_ stimulation could be the reason why Gr21a-OSNs resolved more rapid stimulations as compared to OR- and IR-expressing OSNs (Figure 5). A biphasic response improved pulse resolution in antennal lobe neurons (Lei and Hansson, 1999). Besides the OSN itself, the chemistry of CO_2_ could also contribute to better pulse resolution as it will readily hydrate to bicarbonate (Kwon et al., 2007), and the degree of odor clearing is one of the challenges for resolving rapidly fluctuating odorant stimuli (Ishida and Leal, 2005; Ghatpande and Reisert, 2011).

## Conclusion

Terrestrial olfaction requires the tracking of brief, intermittent airborne stimuli in a turbulent and dynamic environment. Fast reaction times to pockets of clean air are suggested to be behaviorally important for successful and rapid source location; hence, the selection over evolutionary time for sensitive and high-fidelity odor strand detection and resolution in the insect olfactory system is crucial (Baker and Vickers, 1997). Equally, the temporal structure of olfactory information has been shown to be critical for odor coding in a variety of systems (Laurent et al., 2001). Here we show that IR-expressing OSNs are better in detecting long-lasting odor pulses, but they are less sensitive. That could suggest that they are better at close range odor detection where odor-OR interaction time is not a limiting factor (high molecular flux). In contrast OR-expressing neurons are more sensitive and better at resolving brief (low molecular flux) pulsed stimuli. This diversity in temporal characteristics could provide a broad palette of response kinetics for the insect olfactory system to respond to the high-dimensional temporal input found in an insect's odor environment.

IRs are the only receptors found in basal insects and conserved between unicellular and multicellular organisms (Croset et al., 2010). ORs appear to have derived from the gustatory receptor family (Robertson et al., 2003; Nordström et al., 2011), which is present in insects as well as in aquatic arthropods such as water fleas (Peñalva-Arana et al., 2009). Besides increasing the diversity of chemicals that could be detected, OR-OSNs also allow the olfactory system to rapidly detect and transduce brief airborne odor information. This is especially important for flying insects, for which stimulus contact is brief and fast response in time is most critical. OR-expressing OSNs were indeed more sensitive to intermittent stimuli than IRs and Gr21a. The sensitive and fast neuronal response observed in OR-expressing OSNs could result from Orco-dependent transduction, which may have evolved through selective pressure to increase sensitivity and speed of odor detection while in flight.

### Conflict of interest statement

The authors declare that the research was conducted in the absence of any commercial or financial relationships that could be construed as a potential conflict of interest.

## References

[B1] Ai M. Min S. Grosjean Y. Leblanc C. Bell R. Benton R. (2010). Acid sensing by the *Drosophila* olfactory system. Nature 468, 691–695 10.1038/nature09537 21085119 PMC3105465

[B2] Baker T. C. Hansson B. S. Löfsted C. Löfqvist J. (1988). Adaptation of antennal neurons in moths is associated with cessation of pheromone-mediated upwind flight. Proc. Natl. Acad. Sci. U.S.A. 85, 9826–9830 3200859 10.1073/pnas.85.24.9826 PMC282874

[B3] Baker T. C. Vickers N. J. (1997). Pheromone-mediated flight in moths, In Pheromone Research: New Directions. ed Cardé R. T. (Minks AK. New York: Chapman and Hall), 248–264

[B4] Barrozo R. B. Kaissling K.-E. (2002). Repetitive stimulation of olfactory receptor cells in female silkmoths *Bombyx mori* L. J. Insect Physiol. 48, 825–834 10.1016/S0022-1910(02)00109-9 12770060

[B5] Bau J. Justus K. A. Carde' R. T. (2002). Antennal resolution of pulsed pheromone plumes in three moth species. J. Insect Physiol. 48, 433–442 10.1016/S0022-1910(02)00062-8 12770092

[B6] Benton R. Sachse S. Michnick S. W. Vosshall L. B. (2006). Atypical membrane topology and heteromeric function of *Drosophila* odorant receptors *in vivo*. PLoS Biol. 4:e20 10.1371/journal.pbio.0040020 16402857 PMC1334387

[B7] Benton R. Vannice K. S. Gomez-Diaz C. Vosshall L. B. (2009). Variant ionotropic glutamate receptors as chemosensory receptors in *Drosophila*. Cell 136, 149–162 10.1016/j.cell.2008.12.001 19135896 PMC2709536

[B8] Brown S. L. Joseph J. Stopfer M. (2005). Encoding a temporally structured stimulus with a temporally structured neural representation. Nat. Neurosci. 8, 1568–1576 10.1038/nn1559 16222230

[B9] Christensen T. A. Hildebrand J. G. (1997). Coincident stimulation with pheromone components improves temporal pattern resolution in central olfactory neurons. J. Neurophysiol. 77, 775–781 9065849 10.1152/jn.1997.77.2.775

[B10] Clyne P. J. Warr C. G. Freeman M. R. Lessing D. Kim J. Carlson J. R. (1999). A novel family of divergent seven-trans- membrane proteins: candidate odorant receptors in *Drosophila*. Neuron 22, 327–338 10.1016/S0896-6273(00)81093-4 10069338

[B11] Couto A. Alenius M. Dickson B. J. (2005). Molecular anatomical and functional organization of the *Drosophila* olfactory system. Curr. Biol. 15, 1535–1547 10.1016/j.cub.2005.07.034 16139208

[B12] Croset V. Rytz R. Cummins S. F. Budd A. Brawand D. Kaessmann H. (2010). Ancient protostome origin of chemosensory ionotropic glutamate receptors and the evolution of insect taste and olfaction. PLoS Genet. 6:e1001064 10.1371/journal.pgen.1001064 20808886 PMC2924276

[B13] DeBruyne M. Baker T. C. (2008). Odor detection in insects: volatile codes. J. Chem. Ecol. 34, 882–897 10.1007/s10886-008-9485-4 18535862

[B14] Deng Y. Zhang W. Farhat K. Oberland S. Gisselmann G. Neuhaus E. M. (2011). The stimulatory Gαs protein is involved in olfactory signal transduction in *Drosophila*. PLoS ONE 6:e18605 10.1371/journal.pone.0018605 21490930 PMC3072409

[B15] Dolzer J. Fischer K. Stengl M. (2003). Adaptation in pheromone sensitive trichoid sensilla of the hawkmoth *Manduca sexta*. J. Exp. Biol. 206, 1575–1588 10.1242/jeb.00302 12654896

[B16] Ghatpande A. S. Reisert J. (2011). Olfactory receptor neuron responses coding for rapid odour sampling. J. Physiol. 589, 2261–2273 10.1113/jphysiol.2010.203687 21486768 PMC3098702

[B17] Gomez-Marin A. Greg J. Stephens G. J. Louis M. (2011). Active sampling and decision making in *Drosophila* chemotaxis. Nat. Commun. 2, 441 10.1038/ncomms1455 21863008 PMC3265367

[B18] Goulding S. E. zur Lage P. Jarman A. P. (2000). Amos, a proneural gene for *Drosophila* olfactory sense organs that is regulated by lozenge. Neuron 25, 69–78 10.1016/S0896-6273(00)80872-7 10707973

[B19] Gupta B. P. Rodrigues V. (1997). Atonal is a proneural gene for a subset of olfactory sense organs in *Drosophila*. Genes Cells 2, 225–233 9189759 10.1046/j.1365-2443.1997.d01-312.x

[B20] Hallem E. A. Ho M. G. Carlson J. R. (2004). The molecular basis of odor coding in the *Drosophila* antenna. Cell 117, 965–979 10.1016/j.cell.2004.05.012 15210116

[B21] Ishida Y. Leal W. S. (2005). Rapid inactivation of a moth pheromone. Proc. Natl. Acad. Sci. U.S.A. 102, 14075–14079 10.1073/pnas.0505340102 16172410 PMC1216831

[B22] Jones W. D. Cayirlioglu P. Kadow I. G. Vosshall L. B. (2007). Two chemosensory receptors together mediate carbon dioxide detection in *Drosophila*. Nature 445, 86–90 10.1038/nature05466 17167414

[B23] Kaissling K. E. Zack Strausfeld C. Rumbo E. R. (1987). Adaptation processes in insect olfactory receptors. Mechanisms and behavioral significance. Ann. N.Y. Acad. Sci. 510, 104–112 10.1111/j.1749-6632.1987.tb43475.x 3324874

[B24] Kwon J. Y. Dahanukar A. Weiss L. A. Carlson J. R. (2007). The molecular basis of CO_2_ reception in *Drosophila*. Proc. Natl. Acad. Sci. U.S.A. 104, 3574–3578 10.1073/pnas.0700079104 17360684 PMC1805529

[B25] Laurent G. Stopfer M. Friedrich R. W. Rabinovich M. I. Volkovskii A. Abarbanel H. D. (2001). Odor encoding as an active, dynamical process: experiments, computation, and theory. Annu. Rev. Neurosci. 24, 263–297 10.1146/annurev.neuro.24.1.263 11283312

[B26] Lei H. Hansson B. S. (1999). Central processing of pulsed pheromone signals by antennal lobe neurons in the male moth *Agrotis segetum*. J. Neurophysiol. 81, 1113–1122 10085338 10.1152/jn.1999.81.3.1113

[B27] Lei H. Riffell J. A. Gage S. L. Hildebrand J. G. (2009). Contrast enhancement of stimulus intermittency in a primary olfactory network and its behavioral significance. J. Biol. 8, 21 10.1186/jbiol120 19232128 PMC2687775

[B28] Lemon W. C. Getz W. M. (1997). Temporal resolution of general odor pulses by olfactory sensory neurons in American cockroaches. J. Exp. Biol. 200, 1809–1819 9319720 10.1242/jeb.200.12.1809

[B29] Louis M. Huber T. Benton R. Sakmar T. P. Vosshall L. B. (2008). Bilateral olfactory sensory input enhances chemotaxis behavior. Nat. Neurosci. 11, 187–199 10.1038/nn2031 18157126

[B30] Mazor O. Laurent G. (2005). Transient dynamics versus fixed points in odor representations by locust antennal lobe projection neurons. Neuron 48, 661–673 10.1016/j.neuron.2005.09.032 16301181

[B31] Murlis J. Willis M. A. Carde' R. T. (2000). Spatial and temporal structures of pheromone plumes in fields and forests. Physiol. Entomol. 25, 211–222

[B32] Nagel K. I. Wilson R. I. (2011). Biophysical mechanisms underlying olfactory receptor neuron dynamics. Nat. Neurosci. 14, 208–216 10.1038/nn.2725 21217763 PMC3030680

[B33] Nordström K. J. Almen M. S. Edstam M. M. Fredriksson R. Schioth H. B. (2011). Independent HHsearch, Needleman-Wunsch-based and motif analyses reveals the overall hierarchy for most of the G protein-coupled receptor families. Mol. Biol. Evol. 28, 2471–2480 10.1093/molbev/msr061 21402729

[B34] Olsson S. B. Getahun M. N. Wicher D. Hansson B. S. (2011). Piezo-controlled microinjection: an *in vivo* complement for *in vitro* sensory studies in insects. J. Neurosci. Methods 201, 385–389 10.1016/j.jneumeth.2011.08.015 21871493

[B35] Olsson S. B. Kuebler L. S. Veit D. Steck K. Schmidt A. Knaden M. (2011). A novel multi-component stimulus device for use in olfactory experiments. J. Neurosci. Methods 195, 1–9 10.1016/j.jneumeth.2010.09.020 20933006

[B36] Pellegrino M. Nakagawa T. Vosshall L. B. (2010). Single sensillum recordings in the insects *Drosophila melanogaster* and *Anopheles gambiae*. J. Vis. Exp. 17, 1–5 10.3791/1725 20164822 PMC2830253

[B37] Peñalva-Arana D. C. Lynch M. Robertson H. M. (2009). The chemoreceptor genes of the waterflea *Daphnia pulex*: many GRs but no ORs. BMC Evol. Biol. 9:79 10.1186/1471-2148-9-79 19383158 PMC2680840

[B38] Robertson H. M. Warr C. G. Carlson J. R. (2003). Molecular evolution of the insect chemoreceptor gene superfamily in *Drosophila melanogaster*. Proc. Natl. Acad. Sci. U.S.A. 100, 14537–14542 10.1073/pnas.2335847100 14608037 PMC304115

[B39] Sargsyan V. Getahun M. N. Llanos. S. L. Olsson S. B. Hansson B. S. Wicher D. (2011). Phosphorylation via PKC regulates the function of the *Drosophila* odorant coreceptor. Front. Cell. Neurosci. 5:5 10.3389/fncel.2011.00005 21720521 PMC3118453

[B40] Sato K. Pellegrino M. Nakagawa T. Nakagawa T. Vosshall L. B. Touhara K. (2008). Insect olfactory receptors are heteromeric ligand-gated ion channels. Nature 452, 1002–1006 10.1038/nature06850 18408712

[B41] Sato K. Tanaka K. Touhara K. (2011). Sugar-regulated cation channel formed by an insect gustatory receptor. Proc. Natl. Acad. Sci. U.S.A. 108, 11680–11685 10.1073/pnas.1019622108 21709218 PMC3136286

[B42] Silbering A. F. Rytz R. Grosjean Y. Abuin L. Ramdya P. Jefferis G. S. (2011). Complementary function and integrated wiring of the evolutionarily distinct *Drosophila* olfactory subsystems. J. Neurosci. 31, 13357–13375 10.1523/JNEUROSCI.2360-11.2011 21940430 PMC6623294

[B43] Song E. de Bivort B. Dan C. Kunes S. (2012). Determinants of the *Drosophila* odorant receptor pattern. Dev. Cell 22, 363–376 10.1016/j.devcel.2011.12.015 22340498

[B44] Su C. Y. Martelli C. Emonet T. Carlson J. R. (2011). Temporal coding of odor mixtures in an olfactory receptor neuron. Proc. Natl. Acad. Sci. U.S.A. 108, 5075–5080 10.1073/pnas.1100369108 21383179 PMC3064350

[B45a] Theodoni P. Kovacs G. Greenlee M. W. Deco G. (2011). Neuronal adaptation effects in decision making. J. Neurosci. 31, 234–246 10.1523/JNEUROSCI.2757-10.2011 21209209 PMC6622733

[B45] Thorne N. Chromey C. Bray S. Amrein H. (2004). Taste perception and coding in *Drosophila*. Curr. Biol. 14, 1065–1079 10.1016/j.cub.2004.05.019 15202999

[B46] Torre V. Ashmore J. F. Lamb T. D. Menini A. (1995). Transduction and adaptation in sensory receptor cells. J. Neurosci. 75, 7757–7768 8613717 10.1523/JNEUROSCI.15-12-07757.1995 PMC6577959

[B47] Vickers N. J. Christensen T. A. Baker T. C. Hildebrand J. G. (2001). Odour-plume dynamics influence the brain's olfactory code. Nature 410, 466–470 10.1038/35068559 11260713

[B48] Vosshall L. B. Amrein H. Morozov P. S. Rzhetsky A. Axel R. (1999). A Spatial map of olfactory receptor expression in the *Drosophila* antenna. Cell 96, 725–736 10.1016/S0092-8674(00)80582-6 10089887

[B49] Wang Z. Singhvi A. Kong P. Scott K. (2004). Taste representations in the *Drosophila* brain. Cell 117, 981–991 10.1016/j.cell.2004.06.011 15210117

[B50] Wicher D. Schafer R. Bauernfeind R. Stensmyr M. C. Heller R. Heinemann S. H. (2008). *Drosophila* odorant receptors are both ligand-gated and cyclic-nucleotide-activated cation channels. Nature 452, 1007–1011 10.1038/nature06861 18408711

[B51] Yao C. A. Carlson J. R. (2010). Role of G-Proteins in odor-sensing and CO_2_-sensing neurons in *Drosophila*. J. Neurosci. 30, 4562–4572 10.1523/JNEUROSCI.6357-09.2010 20357107 PMC2858456

[B52] Yao C. A. Ignell R. Carlson J. R. (2005). Chemosensory coding by neurons in the coeloconic sensilla of the *Drosophila* antenna. J. Neurosci. 37, 8359–8367 10.1523/JNEUROSCI.2432-05.2005 16162917 PMC6725686

[B53] zur Lage P. I. Prentice D. R. Holohan E. E. Jarman A. P. (2003). The *Drosophila* proneural gene *amos* promotes olfactory sensillum formation and suppresses bristle formation. Development 130, 4683–4693 10.1242/dev.00680 12925594

